# Supplementary material for: A Machine Learning Framework to Predict the Tensile Stress of Natural Rubber: Based on Molecular Dynamics Simulation Data
Source: Polymers (Basel). 2022 May 6;14(9):1897. doi: 10.3390/polym14091897 (PMC9103449; doi:10.3390/polym14091897)
Supplement: Supplementary file 1 [file polymers-14-01897-s001.zip › polymers-1695876-supplementary.pdf]

Supporting Information

A Machine Learning Framework for Predicting the Tensile Stress of Natural Rubber: Based on Molecular Dynamics Simulation Data

Yongdi Huang [1,†], Qionghai Chen [2,†], Zhiyu Zhang [2], Ke Gao [2], Anwen Hu [1], Yining Dong [3,*], Jun Liu [2,*] and Lihong Cui [1,*]

[1] College of Mathematics and Physics, Beijing University of Chemical Technology, Beijing, China
[2] State Key Laboratory of Organic-Inorganic Composites, Beijing University of Chemical Technology, Beijing, China
[3] School of Data Science and Hong Kong Institute for Data Science, Centre for Systems Informatics Engineering, City University of Hong Kong, Tat Chee Avenue, Kowloon, Hong Kong
* Correspondence: liujun@mail.buct.edu.cn (J. Liu); mathcui@163.com (L. H. Cui); yining.dong@cityu.edu.hk (Y. N. Dong)
† These authors contributed equally to this work.

1. K-Means

K-means is a typical algorithm of clustering. It is an unsupervised learning method whose purpose is to divide data into meaningful or useful clusters[1,2]. It was proposed by James in 1967. The basic procedure can be divided into the following parts: First, determine the number of clusters and select k initial centroids. Then calculate the distance between all samples and these k centroids, divide the samples according to the distance, and calculate the new centroid of each cluster. At last, iterate the steps above until the centroids no longer changes.

In this article, we use Euclidean distance to represent the distance from the sample point to the centroid of the cluster where it belongs. The Euclidean distance is described as

$$d(x,\mu) = \sqrt{\sum_{i=1}^{n}(x_i-\mu_i)^2} \qquad (1)$$

where x is a data point in cluster C_k, μ is the centroid of the cluster, n is the number of features in each data points. The cluster sum of squares (CSS) of the distances from all sample points to the centroid is described as

$$CSS = \sum_{j=0}^{m}\sum_{i=1}^{n}(x_i-\mu_i)^2 \qquad (2)$$

where m is the number of clusters. Eq. (2) is also called inertia. The total inertia is the sum of all the CSSs. It can be seen as the "loss function" of a K-means model because the problem is converted into finding the centroids that minimizes the total inertia.

Since it is as unsupervised learning algorithm, the real labels of the samples are unknown. So, the Silhouette Coefficient (SC) is chosen as the evaluation index of K-means. It is also used to select the best number of clusters. The SC value of one single sample is defined as

$$SC = \frac{b-a}{max(a,b)} \qquad (3)$$

where a is the average distance between the data point and all other points in the same cluster, b is the average distance between the data point and all points in the nearest cluster.

Before SMOTE interpolation, cluster analysis of imbalanced data set is required. This study chooses K-means as the cluster algorithm. The key parameter K (the number of clusters) in K-means is determined by repetitive experiments with different K values.

After each iteration, the distribution of the Silhouette Coefficient and the scatter plot of the data set are plotted in Figure S1, Figure S2, Figure S3 and Figure S4.

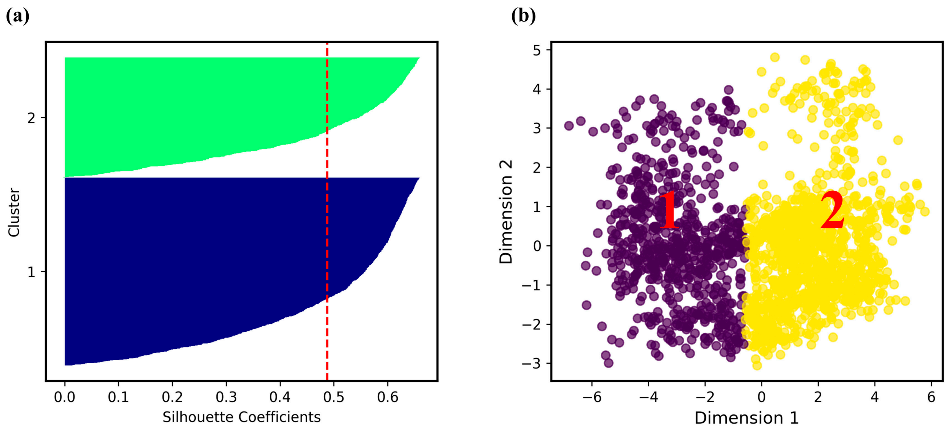

Figure S1. Distribution of the Silhouette Coefficient of each sample and the scatter plot for various clusters when K = 2.

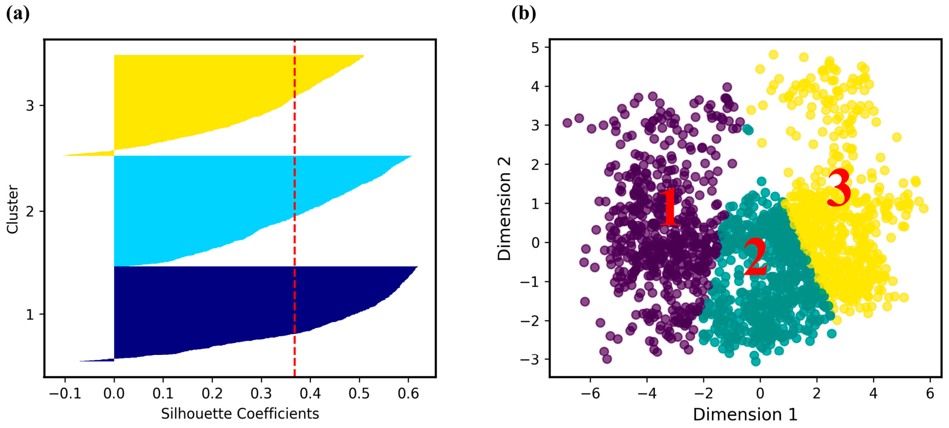

Figure S2. Distribution of the Silhouette Coefficient of each sample and the scatter plot for various clusters when K = 3.

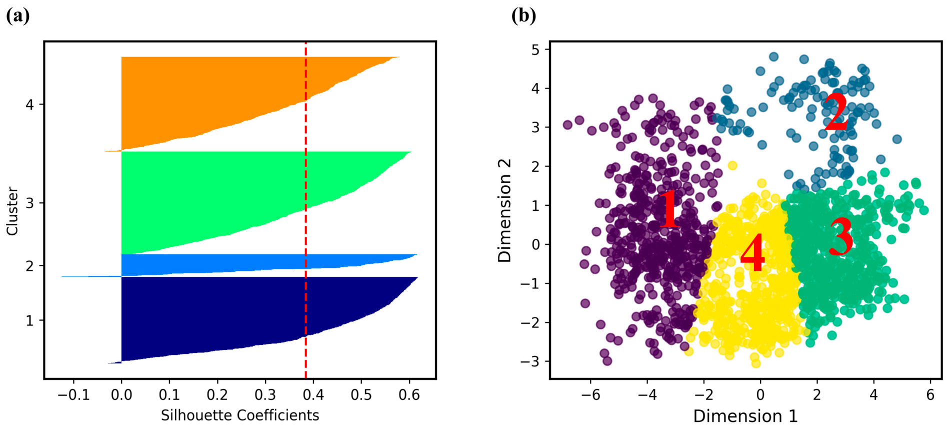

Figure S3. Distribution of the Silhouette Coefficient of each sample and the scatter plot for various clusters when K = 4.

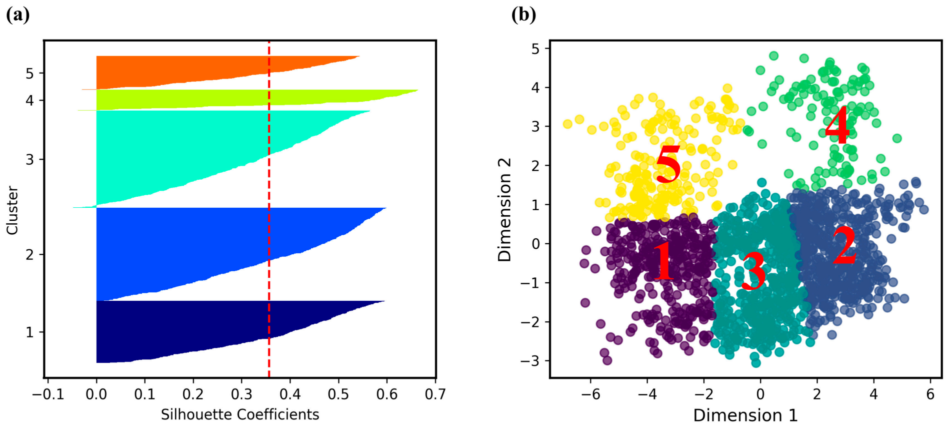

Figure S4. Distribution of the Silhouette Coefficient of each sample and the scatter plot for various clusters when K = 5.

The Silhouette Coefficient plots show the SC score of each sample when the different number of clusters are chosen. Different color means different clusters. Generally speaking, if the SC score of a sample is close to 1, it means that the distance between the sample and the centroid of its cluster is short, and at the mean time far from other clusters' centroids. When K=2, the corresponding average SC score reaches the maximum value 0.488. So, the entire data set will be divided into two clusters.

References

1 Piegl, L. A.; Tiller, W. Algorithm for Finding All k Nearest Neighbors. *Comput. Des.* **2002**, *34*, 167–172.

2 Montavon, G.; Orr, G.B.; Mueller, K.-R. *Neural Networks: Tricks of the Trade*, 2nd ed.; Springer: Berlin, Germany, 2012; pp. 639–655.
